# Histone deacetylase 6 controls Notch3 trafficking and degradation in T-cell acute lymphoblastic leukemia cells

**DOI:** 10.1038/s41388-018-0234-z

**Published:** 2018-04-12

**Authors:** Marica Pinazza, Margherita Ghisi, Sonia Minuzzo, Valentina Agnusdei, Gianluca Fossati, Vincenzo Ciminale, Laura Pezzè, Yari Ciribilli, Giorgia Pilotto, Carolina Venturoli, Alberto Amadori, Stefano Indraccolo

**Affiliations:** 1 Istituto Oncologico Veneto IOV—IRCCS, Padova, Italy; 2 Department of Surgery, Oncology and Gastroenterological Sciences, University of Padova, Padova, Italy; 3 Italfarmaco S.P.A, Milan, Italy; 4 Laboratory of Molecular Cancer Genetics, CIBIO, University of Trento, Trento, Italy; 5 Present Address: CRCT, Toulouse, France

**Keywords:** Target identification, Paediatric cancer, Acute lymphocytic leukaemia, Lysosomes

## Abstract

Several studies have revealed that endosomal sorting controls the steady-state levels of Notch at the cell surface in normal cells and prevents its inappropriate activation in the absence of ligands. However, whether this highly dynamic physiologic process can be exploited to counteract dysregulated Notch signaling in cancer cells remains unknown. T-ALL is a malignancy characterized by aberrant Notch signaling, sustained by activating mutations in Notch1 as well as overexpression of Notch3, a Notch paralog physiologically subjected to lysosome-dependent degradation in human cancer cells. Here we show that treatment with the pan-HDAC inhibitor Trichostatin A (TSA) strongly decreases Notch3 full-length protein levels in T-ALL cell lines and primary human T-ALL cells xenografted in mice without substantially reducing *NOTCH3* mRNA levels. Moreover, TSA markedly reduced the levels of Notch target genes, including *pTα*, *CR2*, and *DTX-1*, and induced apoptosis of T-ALL cells. We further observed that Notch3 was post-translationally regulated following TSA treatment, with reduced Notch3 surface levels and increased accumulation of Notch3 protein in the lysosomal compartment. Surface Notch3 levels were rescued by inhibition of dynein with ciliobrevin D. Pharmacologic studies with HDAC1, 6, and 8-specific inhibitors disclosed that these effects were largely due to inhibition of HDAC6 in T-ALL cells. HDAC6 silencing by specific shRNA was followed by reduced Notch3 expression and increased apoptosis of T-ALL cells. Finally, HDAC6 silencing impaired leukemia outgrowth in mice, associated with reduction of Notch3 full-length protein in vivo. These results connect HDAC6 activity to regulation of total and surface Notch3 levels and suggest HDAC6 as a potential novel therapeutic target to lower Notch signaling in T-ALL and other Notch3-addicted tumors.

## Introduction

T-cell acute lymphoblastic leukemia (T-ALL) is a malignancy of T lymphocytes precursors characterized by a relatively unfavorable prognosis compared to B-cell ALL [1]. Molecular studies uncovered that T-ALL is a disease frequently driven by activating mutations of Notch1, which are found in more than 50% of cases [2]. Although Notch3 mutations are uncommon in patients, Notch3 overexpression is often observed in human T-ALL. Moreover, enforced expression of the active intracellular domain of Notch3 (Notch3-ICD) has been reported to cause T-cell leukemia in mouse models [3, 4]. Given some limitations of existing drugs blocking Notch signaling [5], it is important to get new insights into the biology of Notch3 to further stimulate the development of Notch-targeted therapies in cancer.

Several studies disclosed that ubiquitination and endocytosis regulate activity of both Notch and its ligands [6]. In Drosophila, Notch proteolytic processing is facilitated by dynamin-dependent endocytosis of Notch in the signal-receiving cell [7], with the contribution of the syntaxin Avalanche and the Rab5 GTPase [8]. Activated Notch, together with Notch ligands and non-activated Notch, is subsequently internalized and channeled into early sorting endosomes. In flies, E3 ubiquitin ligases including Nedd4 and Su(dx) sort unstimulated Notch into an endosomal compartment destined for recycling and/or degradation [9]. Steady-state levels of Notch at the surface and pathway activity are modulated by this internalization process, which also limits inappropriate activation of the receptor in the absence of ligands [9]. In fact, trafficking of activated Notch into late endosomal compartments, including multivescicular bodies and degradative lysosomes, is associated with attenuation of Notch signaling [10]. In this regard, Jia et al. [11] reported that Notch3 full-length (FL) and Notch3-ICD are subjected to lysosome-dependent degradation in human cancer cells, suggesting a role of endocytosis in Notch3 degradation and signaling.

Histone deacetylases (HDACs) catalyze epigenetic regulation of chromatin promoting repression of gene expression, and also deacetylate a number of non-histone proteins, thus modulating their function [12]. Unlike other HDACs with predominant chromatin remodeling activity, HDAC6 main targets are cytoplasmic proteins, such as α-tubulin, Hsp90, and cortactin. In particular, HDAC6 inhibition determines increased acetylation of α-tubulin, thus accelerating the association of microtubules with dynein and kinesin and leading to increased routing into early endosomes [13]. Along this line, it was described that epidermal growth factor receptor (EGFR) surface levels are regulated by endocytic trafficking through a mechanism involving HDAC6 and tubulin acetylation [14, 15]. Altogether, these findings support a regulatory role for HDAC6 in endocytic cargo transport of certain transmembrane receptors but whether this might modulate expression of Notch is unknown.

Here, we investigated effects of HDAC inhibitors (HDACi) in T-ALL and found that pharmacologic or genetic inactivation of HDAC6 is followed by increased lysosomal localization of Notch3, which correlates with a reduction in signaling strength. These findings suggest that, in addition to well established approaches such as blocking antibodies and γ-secretase inhibitors [5], targeting HDAC6 is a potential novel strategy to lower Notch3 signaling in T-ALL cells.

## Results

### Trichostatin A downregulates Notch3 protein levels in T-ALL cells

**Fig. 1:**
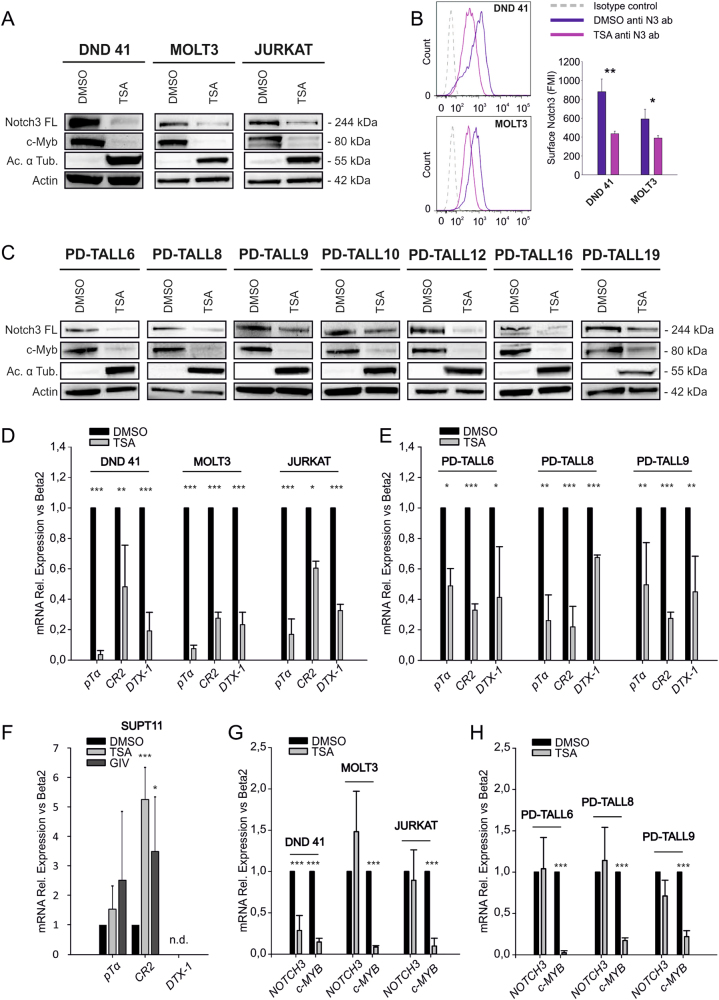
HDAC inhibition reduces Notch3 levels and signaling in T-ALL cells. **a** T-ALL cells (DND 41, MOLT3, and Jurkat) were treated with TSA (0.5 µM) or solvent (DMSO) for 16 h and protein levels analyzed by western blot. Actin was used as a loading control and tubulin acetylation and c-Myb levels as markers of HDAC inhibition. **b** TSA reduces Notch3 surface expression in T-ALL cells. DND 41 and MOLT3 cells treated with TSA or DMSO for 16 h were stained with PE anti-human Notch3 (anti-N3 Ab) or with isotype control antibody and analyzed by flow cytometry. One representative experiment of three performed is shown. Histogram reports fluorescence mean intensity (FMI) ± SD of three independent experiments (**P < 0.01; *P < 0.05). **c** TSA reduces Notch3 expression in PDX-derived T-ALL cells. T-ALL cells obtained from the spleen of xenografted mice were treated in vitro with TSA for 16 h and protein levels were analyzed by western blot. **d-h** Effects of TSA on Notch3 target genes and on Notch transcript levels. T-ALL samples, including both **c**ell lines (**d, f, g**) and PDX T-ALL cells (**e**, **h**), were treated with TSA or Givinostat (GIV) (2 µM) for 16 h and mRNA levels of *NOTCH3, c-MYB*, or Notch target transcripts (*pTα, CR2, DTX-1*) were analyzed by qRT-PCR. n.d.: not detectable. Statistically significant differences are indicated (*P < 0.05, **P < 0.01, ***P < 0.001, mean ± SD of three independent experiments). Expression data are normalized to DMSO samples

Previous studies demonstrated that acetylation regulates Notch3-ICD stability in Notch3 transgenic mice and in one human T-ALL cell line [16]. To validate and broaden these findings, we initially investigated the effects of HDACi on Notch3 in T-ALL cells treated in vitro with the pan-HDAC inhibitor Trichostatin A (TSA) at 0.5 μM, a concentration selected on the basis of published data [16, 17]. After 16 h of treatment, whole-cell lysates were extracted and analyzed by western blot. Accumulation of acetylated α-tubulin and reduction of c-Myb, two known targets of HDACi [17], were used as read-out of TSA activity in these experiments. Interestingly, TSA decreased Notch3 FL levels in all the three cell lines tested (Fig. 1a); Notch3-ICD levels were also reduced (not shown). Following staining with an anti-human Notch3 antibody which binds an extracellular epitope of Notch3, flow cytometry analysis indicated that TSA treatment was followed by reduction of Notch3 surface levels in DND 41 and MOLT3 cells (Fig. 1b). To confirm the results obtained in cell lines, we treated T-ALL cells from several patient-derived xenografts (PDX) with distinct molecular and clinical phenotypes [18]. After 16 h of treatment, Notch3 FL and c-Myb protein levels were strongly reduced in all samples analyzed (Fig. 1c). Importantly, TSA decreased expression of the Notch target genes p*Tα, CR2*, and *DTX-1*, thus suggesting reduction of Notch signaling (Fig. 1d, e), induced apoptosis, and inhibited proliferation of T-ALL cells (Suppl. Figure 1). Effects of TSA on Notch3 levels and signaling were confirmed using the pan-HDAC inhibitor Givinostat, which is currently used in clinical trials (Suppl. Figure 2). Notably, we found no reduction of *pTα* and *CR2* levels in the SUPT11 cell line treated with panHDACi TSA or with Givinostat (Fig. 1f), indicating that HDAC inhibition fails to reduce Notch signaling in cells which do not express detectable Notch3 [19].

To investigate whether these effects were associated with inhibition of transcription, we analyzed mRNA levels of *NOTCH3* and *c-MYB* upon TSA treatment. Interestingly, *c-MYB* mRNA displayed >80% reduction in all samples tested. In contrast, *NOTCH3* transcripts were reduced in DND 41 but not in MOLT3 nor in Jurkat cells (Fig. 1g). Similar results were obtained in three PDX samples (PD-TALL6, PD-TALL8, and PD-TALL9) (Fig. 1h). Altogether, these results indicate that TSA regulates Notch3 expression mainly at post-transcriptional level in the majority of the T-ALL samples analyzed.

### Lysosomal degradation accounts for reduced Notch3 levels in T-ALL cells treated with TSA

**Fig. 2:**
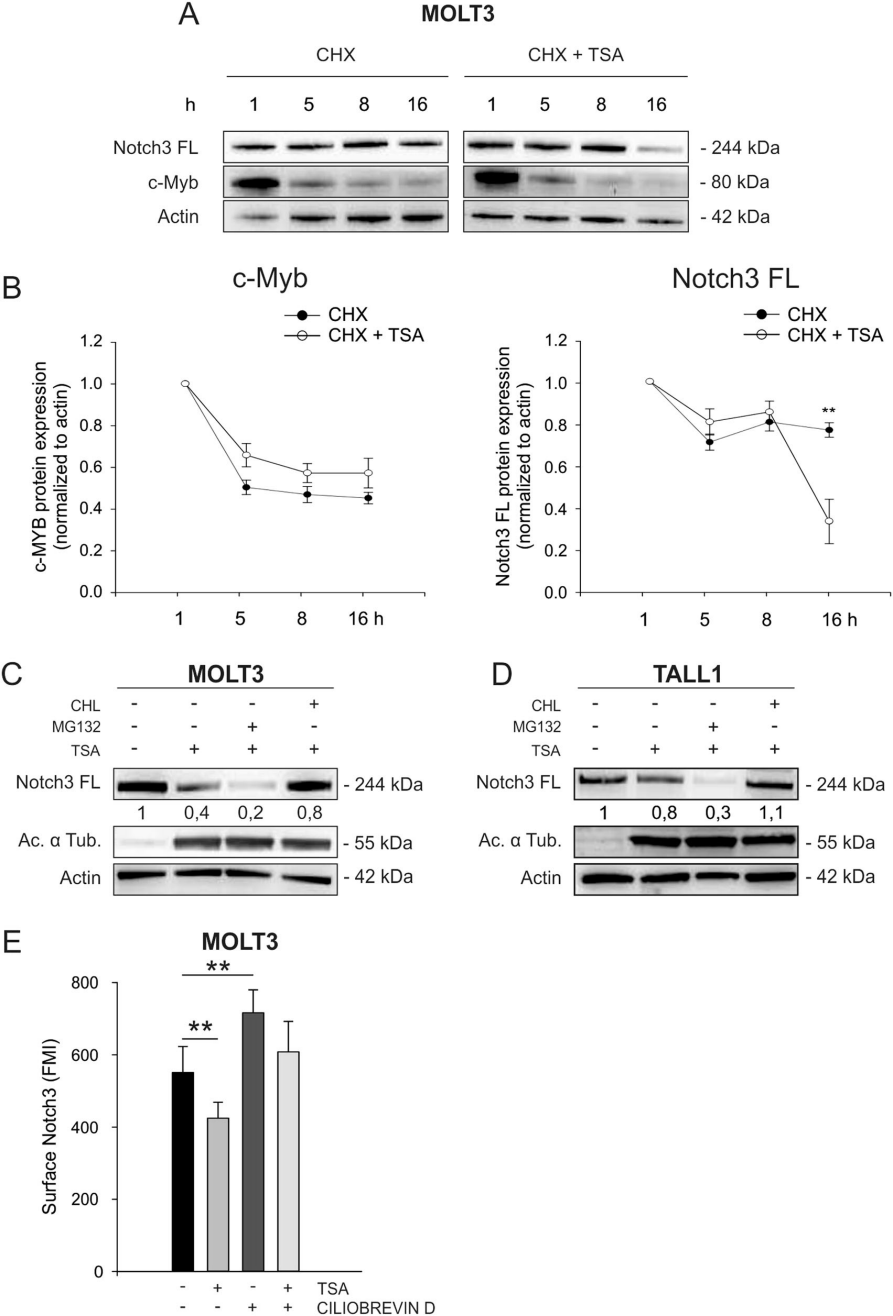
HDAC inhibition promotes Notch3 degradation through the lysosomal pathway. **a** MOLT3 cells were treated with cyclohexymide (CHX, 500 µM) or with CHX plus TSA (0.5 µM). At 1, 5, 8, and 16 h, protein levels of c-Myb and Notch3 FL were analyzed. One representative western blot is reported. **b** c-Myb (left) and Notch3 FL (right) protein expression in three independent experiments was measured by densitometric analysis and normalized to Actin (**P< 0.01). MOLT3 (**c**) or TALL1 cell lines (**d**) were treated with TSA plus MG132 (20 µM) or chloroquine (CHL) (20 µM) for 16 h followed by western blot analysis. Numbers indicate results of densitometric analysis of Notch3 FL bands normalized to Actin. (**e**) MOLT3 were pre-treated with ciliobrevin D (20 µM) for 24 h and then with vehicle or TSA (0.5 µM) for 16 h. Cells were stained with PE anti-N3 antibody and analyzed by FACS analysis. Statistically significant differences are indicated (**P < 0.01, mean ± SD of three independent experiments)

**Fig. 3:**
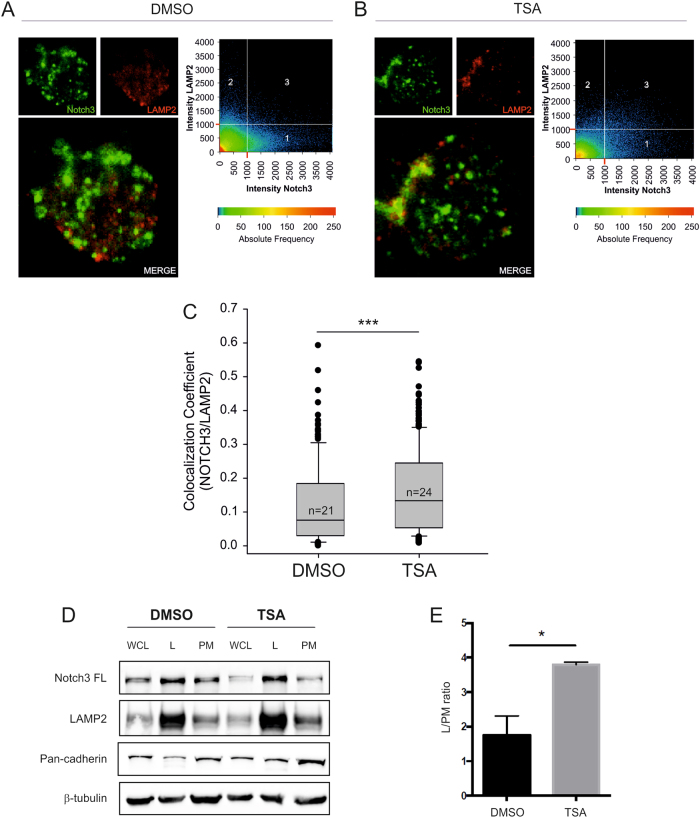
TSA increases co-localization of Notch3 protein with LAMP2-positive vesicles and increases the abundance of Notch3 FL in the lysosomal compartment. MOLT3 cells were treated with DMSO or TSA (0.5 µM) for 8 h and were subsequently immuno-stained for Notch3 and LAMP2. *n* = 21 DMSO-treated cells and *n* = 24 TSA-treated cells were analyzed by z-stack laser scanning microscopy using a ×63 oil objective. Images were acquired with a resolution of 1024 × 1024 pixels. **a, b** Representative optical slices taken in the apical portion of the cells above the nucleus are shown. Scatterplots on the right-hand portion of the panels indicate the fluorescence intensity of Notch3 (*X-*axis) and LAMP2 (*Y-*axis) detected in each pixel. Detection thresholds were set at 1000 for both channels. Region 1: Notch3 single positive pixels. Region 2: LAMP2 single positive pixels. Region 3 double-positive (Notch3/LAMP2) pixels. **c** Co-localization coefficients were calculated in 21 mock-treated cells and 24 TSA-treated cells using the Zeiss Histogram software tool. At least 15 optical slices were analyzed for each cell. The values of the co-localization coefficients range between 0 and 1. Box plots reported medians, lower/upper quartiles, and outliers of all co-localization coefficients obtained for DMSO and TSA cells respectively. *** indicates *P* < 0.001 (Mann–Whitney test). **d** DND 41 cells were treated with DMSO or TSA (0.5 µM) for 8 h and 1 × 10^8^ pelleted cells underwent subcellular fractionation. Protein extracts for each fraction were analyzed by western blot. A representative blot is reported. WCL whole-cell lysate; L lysosome; PM plasma membrane. **e** Histogram reports the quantified ratio of lysosomal enriched Notch3 FL normalized to Notch3 FL present on the plasma membrane. Expression of Notch3 FL in the different fractions was normalized to each fraction-specific proteins. Mean ± SD of two independent replicates (*P < 0.05, Student’s *t*-test)

Several reports indicate that HDACi induce degradation of oncogenes and other cellular proteins by affecting protein stability [20]. To test whether protein degradation has a role in the effects of TSA on Notch3 protein levels, we inhibited protein translation in MOLT3 cells with cycloheximide. As expected, based on the fact that HDACi control c-Myb levels mainly at the transcriptional level (Fig. 1g and [17]), the half-life of c-Myb, roughly 8 h in MOLT3 cells, was not substantially changed by TSA. In contrast, Notch3 protein levels decreased faster in the presence of TSA (Fig. 2a, b). This result shows that TSA affects Notch3 protein stability, implying a post-translational mechanism of regulation. To investigate the molecular mechanism underlying increased Notch degradation, we treated MOLT3 and TALL1 cells with TSA in the presence of proteasome or lysosome inhibitors. Notch3 levels were rescued using the lysosome inhibitor chloroquine (CHL), suggesting involvement of the endocytic pathway. In contrast, the proteasome inhibitor MG132 further reduced Notch3 FL levels (Fig. 2c, d), whereas it increased c-Myc protein levels (Suppl. Figure 3), a transcription factor known to be degraded by the proteasome [21, 22]. Similar results were obtained in MOLT3 cells by using bafilomycin as alternative lysosome inhibitor (Suppl. Figure 4). Moreover, treatment with ciliobrevin D, a dynein inhibitor, rescued Notch3 surface levels upon TSA treatment in MOLT3 cells (Fig. 2e), confirming the importance of tubulin acetylation and vesicle transport through cytoplasmic dynein of Notch3 from the cell membrane to the lysosome. In addition, immunofluorescence and confocal microscopy analysis confirmed that MOLT3 cells treated with TSA displayed increased co-localization of Notch3 and the lysosomal marker LAMP2 (Fig. 3a–c). Fractionation assays corroborated these findings by showing that Notch3 was mainly enriched in the lysosomal fraction in T-ALL cells and upon TSA treatment there was a significant increase in the lysosome/plasma membrane ratio (Fig. 3d, e and Suppl. Figure 5). Taken together, these findings indicate that HDAC inhibition results in the accumulation of Notch3 in the lysosomal compartment.

### HDAC6 modulates Notch3 expression and signaling in vitro

**Fig. 4:**
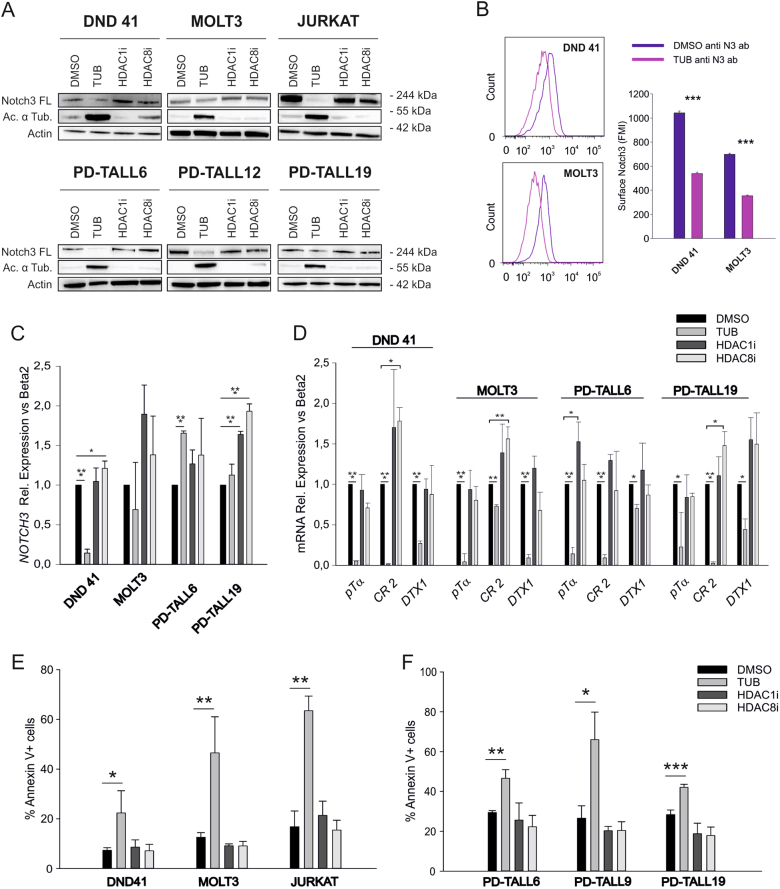
Pharmacologic inhibition of HDAC6 lowers Notch3 FL protein levels and signaling, triggering apoptosis of T-ALL cells. **a** T-ALL cell lines (up) and PDX T-ALL cells (bottom) were treated in vitro for 16 h with the HDAC6 inhibitor tubacin (TUB, 2 µM), HDAC1 inhibitor (HDAC1i, 2 µM), or HDAC8 inhibitor (HDAC8i, 2 µM). Protein levels were analyzed by western blot. **b** Tubacin reduces Notch3 surface expression in T-ALL cells. DND 41 and MOLT3 cells treated with tubacin (2 µM) or DMSO for 16 h were stained with PE anti-human Notch3 (anti-N3 ab) and analyzed by flow cytometry. One representative experiment of three performed is shown. Histogram reports fluorescence mean intensity (FMI) ± SD of three independent experiments (***P < 0.001). Expression levels of *NOTCH3* (**c**) and Notch target genes (**d**) in T-ALL cells treated as above were analyzed by qRT-PCR (*P < 0.05, **P < 0.01, ***P < 0.001, mean ± SD of three independent experiments). Expression data are normalized to DMSO samples. Induction of apoptosis by HDACi in T-ALL cells (**e**) and in PDX cells (**f**) was measured by flow cytometric analysis of Annexin V staining (*P < 0.05, **P < 0.01, ***P < 0.001, mean ± SD of three independent experiments)

To identify HDAC family member(s) responsible for the effects previously characterized, we tested class-specific HDACi. In particular, we used the HDAC6 inhibitor tubacin, and two HDAC1 and HDAC8 inhibitors. Interestingly, HDAC1i and HDAC8i did not reduce Notch3 FL protein levels or Notch target genes expression and did not exert apoptotic effects in T-ALL cell lines and PDX cells (Fig. 4). On the contrary, the HDAC6-specific inhibitor tubacin reproduced effects of TSA, including reduction of Notch3 surface levels (Fig. 4), suggesting a role of this specific HDAC in the phenomenon observed. To verify activity of these compounds, we also analyzed histone 3 acetylation levels in MOLT3 and Jurkat cells. As expected, tubacin did not change histone acetylation, whereas treatment with HDAC1 and HDAC8 inhibitors increased histone 3 acetylation (Suppl. Figure 6).

**Fig. 5:**
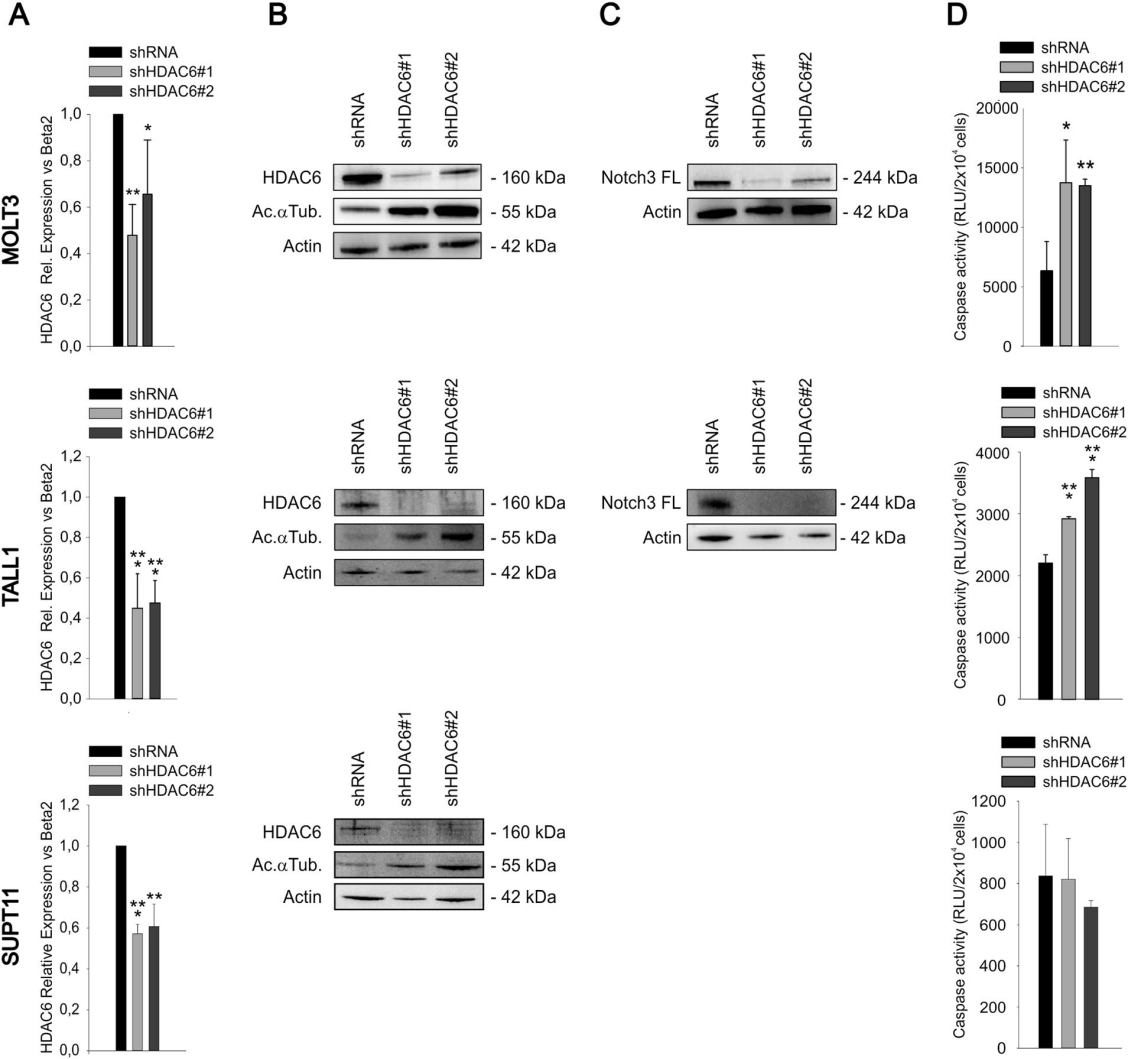
Effects of HDAC6 silencing on Notch3 FL protein levels and apoptosis of T-ALL cells. MOLT3 (top), TALL1 (middle), or SUPT11 (bottom) cells were transduced with lentiviral vectors encoding a scramble shRNA or two different shHDAC6 vectors. qRT-PCR (**a**) and western blot analysis (**b**) performed 96 h after transduction, confirmed the efficacy of HDAC6 silencing by both constructs. *HDAC6* mRNA levels were normalized setting at one the shRNA sample. **c** MOLT3 or TALL1 cells expressing shHDAC6 #1 or shHDAC6 #2 showed reduced Notch3 FL protein levels. Western blot analysis was not performed in SUPT11 cells as it is known that these cells do not express detectable Notch3 [19]. **d** HDAC6 silencing was associated with increased apoptosis of T-ALL cells which express Notch3, but not in SUPT11 cells. Apoptosis was analyzed by caspase 3–7 assay 5 days after transduction of cells with the indicated LV. Relative Luminescence Units (RLU) are reported (*P < 0.05; **P < 0.01, ***P < 0.001, mean ± SD, three independent experiments)

Finally, HDAC6 silencing by two different shRNA in MOLT3 and TALL1 cells was followed by reduced Notch3 FL protein levels (Fig. 5a–c) and induced apoptosis in T-ALL cells (Fig. 5d), thus mimicking effects obtained with tubacin and TSA. Apoptosis induction was not observed in SUPT11 cells, which lack detectable Notch3 (Fig. 5d). Interestingly, HDAC6-mediated regulation of Notch3 FL protein seems specific for this NOTCH paralog, since HDAC6 silencing did not reduce Notch1 FL levels in MOLT3 cells (Suppl. Fig. 7).

### Givinostat and HDAC6 silencing impair Notch3 expression and leukemia growth in vivo

**Fig. 6:**
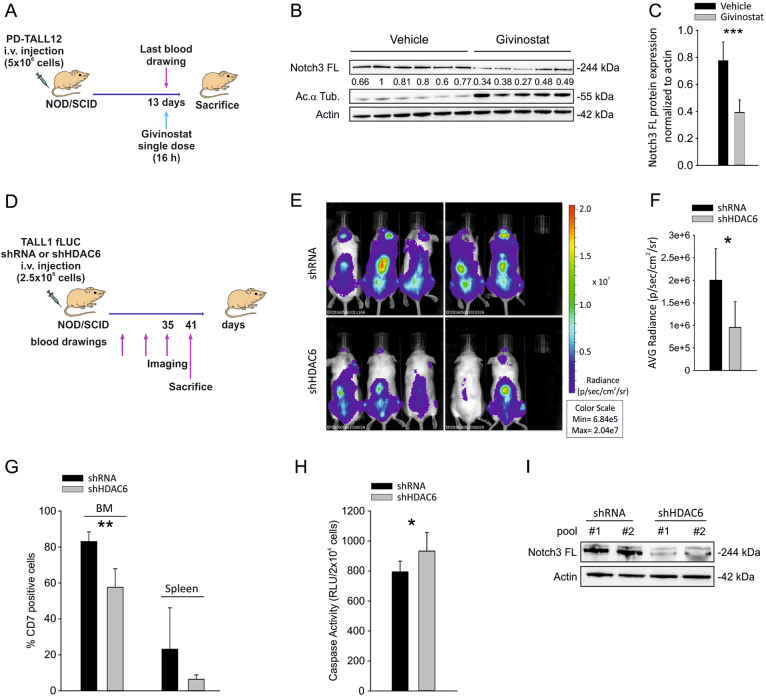
Pharmacologic or genetic HDAC6 inhibition is associated with reduced Notch3 protein levels in vivo and impaired tumor growth. **a** Outline of treatment. PD-TALL12 xenografted NOD/SCID mice were randomized to receive either Givinostat (25 mg/kg) or vehicle i.p. and sacrificed 16 h after treatment. **b** Leukemic cells were recovered from the spleen of PD-TALL12 mice and Notch3 FL and acetylated α-tubulin protein levels analyzed by WB. Numbers indicate results of densitometric analysis of Notch3 FL bands normalized to Actin. **c** Columns report the mean values ± SD of Notch3 to actin ratios (densitometric analysis) in control and treated mice (***P < 0.001). **d** TALL1 cells were serially transduced with a lentiviral vector encoding the Firefly luciferase gene (fLUC) and with lentiviral vectors expressing either a scramble shRNA or an HDAC6-specific shRNA (shHDAC6 #1). Cells were i.v. injected in NOD/SCID mice (2.5 × 10^6^ cells/mouse, *n* = 5 mice/group) and tumor growth was monitored by optical imaging (**e**, **f**). Representative images (**e**) and quantitative analysis (**f**) of luciferase activity at day 35 from TALL1 cells injection. Statistically significant differences in average radiance in the two groups of samples are indicated (*P < 0.05, mean ± SD, *n* = 5 mice/group). **g** Flow cytometric analysis of CD7^+^ cells in the BM and spleen of shRNA and shHDAC6#1 mice at sacrifice (41 days) (**P < 0.01, mean ± SD, *n* = 5 mice/group). **h** Apoptosis was analyzed by caspase 3–7 assay in CD7^+^ cells sorted from the spleen of mice at sacrifice (*P < 0.05, mean ± SD, *n* = 5 mice/group). **i** After sorting, T-ALL cells obtained from different mice were pooled and Notch3 FL protein levels analyzed by western blotting

To investigate whether modulation of Notch3 following treatment with an HDACi occurred in vivo, we treated PD-TALL12 xenografted NOD/SCID mice (*n* = 5/6 per group) with Givinostat (25 mg/kg), a panHDACi used in clinical trials, or PEG400/H_2_0 (vehicle). The drug was administered as a single dose upon establishment of the leukemia in the mice and animals were killed 16 h after treatment (Fig. 6a). At the time of administration of the drug, spleen and bone marrow (BM) infiltration by T-ALL cells was very high and comparable between treated and untreated mice (Suppl. Fig. 8A, B). Western blot analysis of T-ALL cells from the spleen of Givinostat-treated mice or controls showed significantly decreased Notch3 FL protein levels (Fig. 6b, c). Although survival was not an endpoint of this experiment, in our previous study we found that repeated Givinostat administration can extend survival of mice engrafted with PD-TALL12 cells [23].

Pharmacologic issues, in particular the low solubility of tubacin, prevented us from performing this experiment in mice. Therefore, we investigated whether genetic HDAC6 inactivation would impair tumor growth by HDAC6 silencing in the Notch3-dependent cell line TALL1. To this end, we transduced TALL1 cells with a lentiviral vector encoding the firefly luciferase gene (fLUC), in order to track leukemia progression in vivo by optical imaging. TALL1 fLUC cells were then infected with shRNA or with shHDAC6 #1 and injected in NOD/SCID mice (Fig. 6d). Results show significant reduction in leukemia burden in shHDAC6#1 compared to shRNA mice, measured by optical imaging (Fig. 6e, f). At sacrifice, we measured reduction of human CD7-positive cells in the BM and in the spleen (Fig. 6g), induction of apoptosis (Fig. 6h) and reduction of Notch3 FL protein in the spleen of mice injected with HDAC6-silenced T-ALL cells (Fig. 6i). Overall, these results show that inhibition of HDAC6 activity exerts anti-leukemia effects in mice.

## Discussion

Notch is constitutively internalized and it has been proposed that endocytosis modulates Notch signaling both by controlling the amount of surface levels of the receptor and by regulating its activation [24–26]. However, it has been debated whether endocytosis is required for full proteolytic processing of Notch, whereas it seems fundamental to downregulate signaling activity by lowering the potential for Notch interactions with its ligands at the cell surface (reviewed in [27]). Consistent with this model, Notch-signaling defects observed upon disruption of the activity of certain endocytosis-associated molecules, such as clathrin or dynamin [28, 29], may derive from insufficient surface Notch levels rather than selective disruption in receptor internalization. Defects in recycling or in the biosynthetic pathway might be associated with abnormal accumulation of Notch in certain intracellular compartments, such as Golgi [30].

These concepts stemmed from studies carried out in invertebrates, while there is limited information about mechanisms involved in Notch trafficking in human cancer cells, whose dysregulation might perturb Notch homeostasis leading to alterations in signaling. Conversely, interventions on Notch trafficking might represent a new therapeutic strategy to counteract aberrant Notch signaling in cancer.

Here, we describe a novel HDAC-mediated mechanism of regulation of Notch3 involving the lysosomal pathway in T-ALL cells. We report for the first time that HDAC inhibition leads to increased accumulation of Notch3 in lysosomes, reducing total and surface Notch3 levels. Our conclusions are supported by (I) reversion of HDACi effects on Notch3 levels by two different lysosome inhibitors and by blocking dynein function and (II) increased co-localization of Notch3 and the lysosomal marker LAMP2 in T-ALL cells treated with TSA by immunofluorescence studies and fractionation assays. Furthermore, involvement of HDAC6 in the control of Notch3 degradation was shown by both pharmacological inhibition and gene silencing experiments. Our findings are fitting those of previous works that demonstrated increased degradation of EGFR by the endocytic compartment following pharmacologic or genetic inactivation of HDAC6 [14, 15]. In these studies, increased microtubule acetylation accelerated lysosomal accumulation of EGFR-bearing vesicles by an HDAC6-mediated mechanism [14, 15]. We speculate that HDAC6 could also affect Notch3-FL trafficking by indirect mechanisms, namely by modulation of α-tubulin acetylation. This hypothesis stems from the above quoted studies concerning EGFR and the apparent lack of direct interactions between HDAC6 and Notch3 by immunoprecipitation studies (not shown).

Although several studies previously reported pro-apoptotic effects of HDACi in leukemia cells [reviewed in [5]], to our knowledge only one study investigated effects of HDACi on Notch3 levels. Palermo et al. [16] demonstrated that acetylation controls Notch3 stability and function in murine T-ALL cells. In this paper, Notch3-ICD acetylation increased following HDAC1 inhibition, leading to Notch3 increased ubiquitination and proteasome-dependent degradation. However, in our experiments the proteasome inhibitor MG132 did not rescue Notch3 FL degradation, indicating that increased proteosomal degradation does not account for TSA effects on Notch3 FL levels in human T-ALL cells. In fact, MG132 appeared to further reduce Notch3 FL levels when combined with TSA (Fig. 2c, d). Although further studies are needed to understand the molecular basis of this interaction, it has been reported that some proteasome inhibitors interact with dynein and up-regulate endocytosis [31], thus modulating the same biological process as HDAC6 inhibitors. Finally, Notch signaling was not impaired in T-ALL cells following treatment with an HDAC1-specific inhibitor, suggesting that the mechanism proposed by Palermo et al. did not explain results obtained in our experiments.

Notably, shRNA against HDAC6 did not reduce Notch1 levels in MOLT3 cells (Suppl. Fig. 7). Although the reason behind this remains unknown, given the similar molecular structure of Notch1 and Notch3, this finding might underscore differential modalities of degradation of Notch1 compared with Notch3 receptors. In this regard, it is established that Notch1 undergoes ubiquitination and subsequent degradation through the proteasome by FBW7/Itch [32]. In contrast, Jia et al. [11] reported that Notch3 FL and intracellular domain are mainly subjected to lysosome-dependent degradation in various tumor cell lines. Thus, alternative routes of intracellular degradation might be involved in turnover of Notch1 and Notch3 receptors. In any case, persisting Notch1 signaling in T-ALL cells undergoing marked attenuation of Notch3 levels could partially rescue expression of Notch target genes following HDAC6 silencing in T-ALL cells, and shield these cells from the negative consequences of Notch signaling blockade, such as apoptosis or cell cycle arrest. Speculatively, treatment with HDAC6i might exert stronger therapeutic effects in tumor cells bearing Notch3 mutations in the absence of Notch1 mutations, such as the TALL1 cell line. In conclusion, our study disclosed that HDAC6 controls trafficking and lysosomal degradation of Notch3, by a mechanism likely involving acetylation of α-tubulin. Inhibition of HDAC6 with selective drugs may thus represent a new therapeutic approach for Notch3-addicted malignancies.

## Materials and methods

### In vivo experiments

PDX were established in NOD/SCID mice as previously described [18]. PDX growth was monitored using flow cytometry by testing the % of human CD5 and CD7 in blood as reported elsewhere [18]. In a set of experiments, PDX mice (at least 5 per group) were treated with Givinostat (25 mg/kg) or vehicle (PEG400/H_2_0) as a single dose upon establishment of the leukemia and mice were euthanized 16 h later.

In another set of experiments, TALL1 cells were serially transduced with luciferase (fLUC) encoding lentiviral vectors expressing either a HDAC6-specific shRNA or a scramble shRNA as a control and their growth in mice was monitored as previously described [33].

### Cell lines

MOLT3 and Jurkat cell lines were purchased from ATCC (Manassas, VA, USA); DND 41 and TALL1 cell lines were kindly provided by academic colleagues; SUPT11 cell lines were purchased from DSMZ (Deutsche Sammlung von Mikroorganismen und Zellkulturen GmbH, Braunschweig, Germany); all these cell lines were cultured in complete RPMI medium, as reported elsewhere [33]. Cells were periodically tested for mycoplasma contamination. PDX cells were cultured in MEMα medium (Thermo Fisher Scientific) supplemented with 10% human heat inactivated AB^+^ serum, 10% fetal calf serum (FCS), 1% Glutamax, 1% penicillin/streptomycin and with 20 ng/ml FLT3 ligand, 10 ng/ml IL7, 50 ng/ml SCF (Peprotech, Rocky Hill, NJ, USA), and 20 nM human insulin (Sigma Aldrich, Saint Luis, MO, USA).

The following drugs were used: 0.5 µM TSA (Sigma Aldrich), 500 µM cyclohexamide (Sigma Aldrich), 20 µM MG132 (Sigma Aldrich), 20 µM CHL (Sigma Aldrich), 2 µM tubacin (Enzo Life Science, Farmingdale, NY), 100 nM bafilomycin (Sigma Aldrich), 2 µM HDAC1i and HDAC8i (Italfarmaco, Milan, Italy), 20 µM Ciliobrevin D. At planned time points, cells were harvested and processed for assessment of cell viability, caspase assay, and RNA and protein extraction.

### Flow cytometry

Detection of PDX cells in mouse samples was carried out with anti-human FITC-conjugated CD5 and PE-Cy5-conjugated CD7 antibodies (Coulter, Fullerton, CA, USA). Apoptosis was measured using Annexin V marker, as reported [23]. Surface Notch3 levels were analyzed using a PE anti-human Notch3 antibody (Biolegend, San Diego, CA, USA). Samples were analyzed on a Beckman Coulter EPICS-XL Flow Cytometer (Coulter) or a BD LSRII Flow Cytometer (BD Biosciences, San Jose, CA, USA).

### Transduction with lentiviral vectors

Lentiviral vectors encoding shRNA targeting human HDAC6 (Sigma Aldrich; shHDAC6 #1: TRCN0000314909; shHDAC6 #2: TRCN0000314976) or the control shRNA vector were produced and titrated as previously reported [34].

### Reverse transcription-PCR and quantitative PCR (qPCR)

Total RNA was purified by standard procedures as reported elsewhere [23]. Quantitative PCRs of cDNAs were performed with an ABI Prism 7900HT Sequencer (Thermo Fisher Scientific), as described [23]. Primer sequences are shown in Suppl. Table 1.

### Immunoblot analysis

Western blot protocols have been previously published [23]. Immunoprobing was performed using the following antibodies: mouse anti-acetylated α-tubulin (Santa Cruz Biotechnologies, Dallas, TX, USA), rabbit anti-actin (Sigma Aldrich), rabbit anti-Notch3 (Abcam, Cambridge, UK), rabbit anti-Notch1 (Cell Signaling), mouse anti-c-Myb (Thermo Fisher Scientific), rabbit anti-HDAC6 (Santa Cruz Biotechnologies), rabbit anti-Histone H3 (Cell Signaling or Abcam), rabbit anti-acetyl-Histone H3 (Lys 9) (Cell Signaling), mouse anti-LAMP2 (Novus Biologicals, Littleton, CO, USA), mouse anti-Pan-cadherin (Santa Cruz Biotechnologies), mouse anti-GAPDH (Santa Cruz Biotechnologies), rabbit anti-VDAC (Cell Signaling) followed by incubation with a horseradish peroxidase-conjugated anti-rabbit or anti-mouse secondary Ab (Perkin Elmer, Waltham, MA, USA). Antigens were identified using Western Lightning plus ECL (Perkin Elmer) or ECL Select Western Blotting detection (GE Health Care) reagents and detected by UVITec Alliance LD2 (UVITec Cambridge, UK) imaging system.

### Immunofluorescence analysis

The protocol for immunofluorescence analysis has been reported elsewhere [35]. Primary antibodies used included anti-Notch3 mAb (1:100; Abcam) and anti-LAMP2 mAb (1:150; Novus Biologicals, Littleton, CO, USA), both incubated overnight at 4 °C. Confocal microscopy was carried out on a Zeiss LSM 510 microscope (Zeiss, Jena, Germany) using a ×63 oil immersion objective (NA = 1.4). *n* = 21 DMSO-treated cells and *n* = 24 TSA-treated cells were analyzed by z-stack scanning, with an optical slice of 1 μm and average of 15 optical slices/cell. Images were acquired with a resolution of 1024 × 1024 pixels. The co-localization coefficient (which indicates the extent of localization of Notch3 protein in LAMP2-positive vesicles) was calculated, using the Zeiss Histogram software tool, as the ratio between the Notch3/LAMP2 double-positive pixels and total number of Notch3-positive pixels.

### Subcellular fractionation

Subcellular fractionation was performed by differential centrifugation in isotonic sucrose buffer with minor modification to previously described protocols [36]. Briefly, 1 × 10^8^ pelleted cells were resuspended in 1 ml of ice-cold sucrose buffer (0.25 M sucrose in 10 mM Tris-HCl pH 7.4 and Protease Inhibitor Cocktail—Roche Diagnostic) and homogenized for 4 min (10 s ON–10 s OFF) using Kimble Kontes Pellet Pestle (Thermo Fisher Scientific). Homogenized cells were centrifuged at 4 °C at increasing speed to isolate specific cellular compartments, as schematized in Suppl. Figure 5. Pellets were resuspended in sucrose buffer and proteins were quantified using Pierce BCA Protein Assay Kit (Thermo Fisher Scientific). Twenty-five micrograms of protein extracts for each fraction were then solubilized with 3× Laemmli Sample Buffer with 0.1 M DTT, denaturated and analyzed by western blot using 7.5% or 4–15% polyacrylamide gels (BioRad, Munich, Germany). LAMP2, Pan-cadherin, GAPDH, VDAC, Histone H3, and β-tubulin were used as specific markers for lysosomes, plasma membranes, cytosol, mitochondria, nuclei, and total fractions, respectively.

### Proliferation assay

Proliferation of T-ALL cells after HDAC inhibition was measured by the CellTiter 96^®^ AQueous One Solution Cell Proliferation Assay (Promega, Madison, WI, USA).

### Caspase activity assay

Caspase 3–7 activity was evaluated with the CaspaseGlo 3/7 assay kit according to the manufacturer’s recommendations (Promega).

### Statistical analysis

Results were expressed as mean ± standard deviation (SD). Statistical analysis was performed using Student’s *t*-test or the non-parametric Mann–Whitney test, depending on the distribution of values. Differences were considered statistically significant when *P* < 0.05.

## Electronic supplementary material

Supplemental material_clear(DOCX 1061 kb)
