# Supplementary material for: Histone deacetylase 6 controls Notch3 trafficking and degradation in T-cell acute lymphoblastic leukemia cells
Source: Oncogene. 2018 Apr 12;37(28):3839–51. doi: 10.1038/s41388-018-0234-z (PMC6041259; doi:10.1038/s41388-018-0234-z)
Supplement: Supplementary file 1 — Supplemental material_clear(DOCX 1061 kb) [file 41388_2018_234_MOESM1_ESM.docx]

**SUPPLEMENTARY DATA**

| **Suppl. Table 1** |  |
| --- | --- |
|  |  |
| **Primers used for qRT-PCR analysis** | |
| *hC-MYB-*for | 5’-ACCTAGCCCAAGGGTGAACA-3’ |
| *hC-MYB -*rev | 5’-TCGAAGGATGACCAGTGGAA-3’ |
| *hCR2-*for | 5'-CTGCGGTTCAGTGTCCACAT-3' |
| *hCR2-*rev | 5'-GGTGAAGCCAAACATGCAAGC-3' |
| *hDTX1-*for | 5'-GTGGGCTGATGCCTGTGAAT-3' |
| *hDTX1-*rev | 5'-CGAGCGTCCTCCTTCAGCAC-3' |
| *hNOTCH3-*for | 5’-CAAGGGTGAGAGCCTGATGG-3’ |
| *hNOTCH3-*rev | 5’-GAGTCCACTGACGGCAATCC-3’ |
| *hpTα-*for | 5’- ATGGTGGTGGTCTGCCTGGT-3’ |
| *hpTα-*rev | 5’-AGTTGGTCCAGGTGCCATCC-3’ |
| *hHDAC6-*for | 5’-GGCTTCAGTTTCCTGTGCTC-3’ |
| *hHDAC6-*rev | 5’-CTTCCTCCTCGCTCTCCTCT-3’ |
| *h*β*2-microglobulin-*for | 5'-TGCTGTCTCCATGTTTGATGTATCT-3' |
| *h*β*2-microglobulin-*rev | 5'-TCTCTGCTCCCCACCTCTAAGT-3' |

**Suppl. Figure 1**


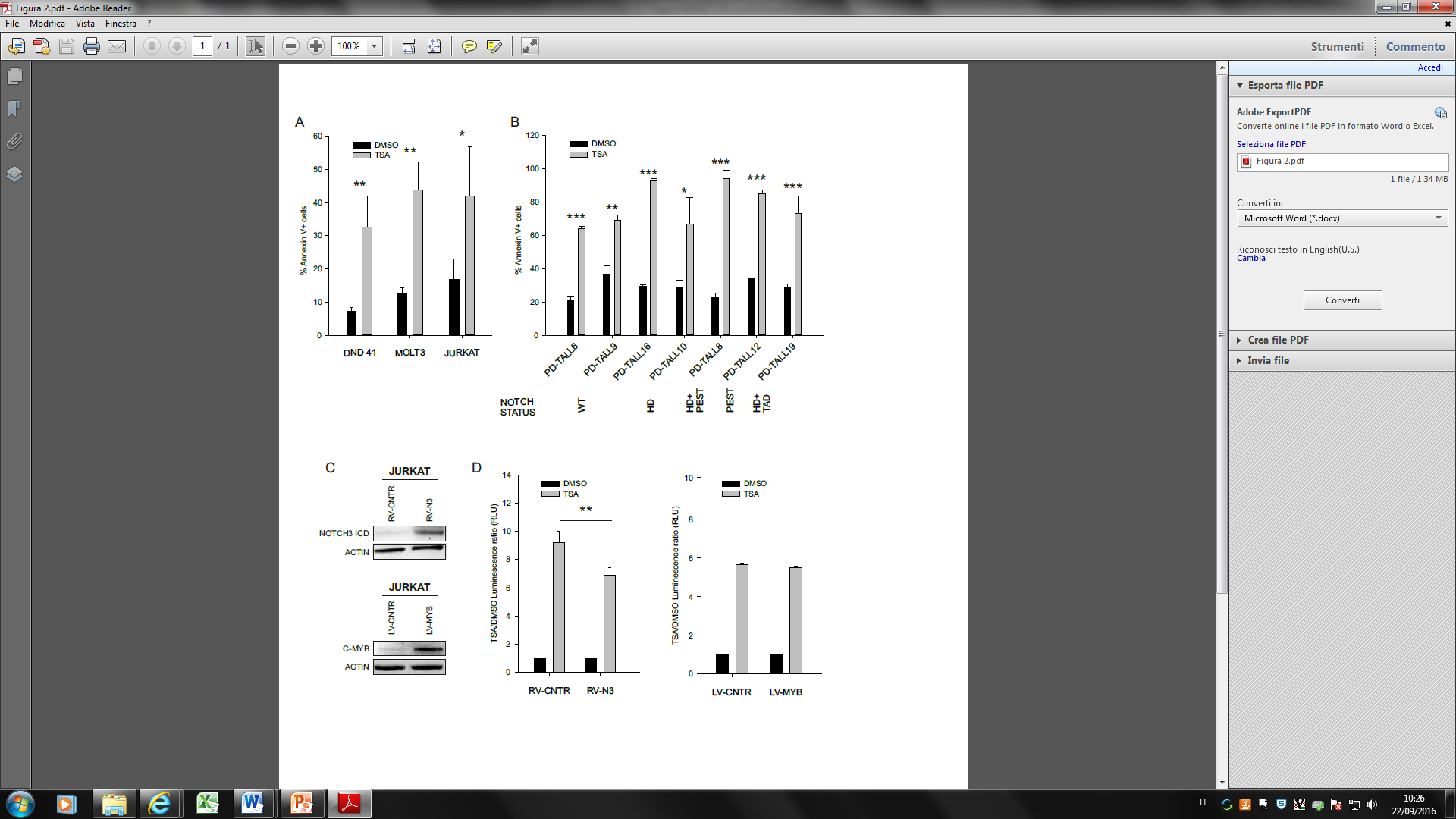


**
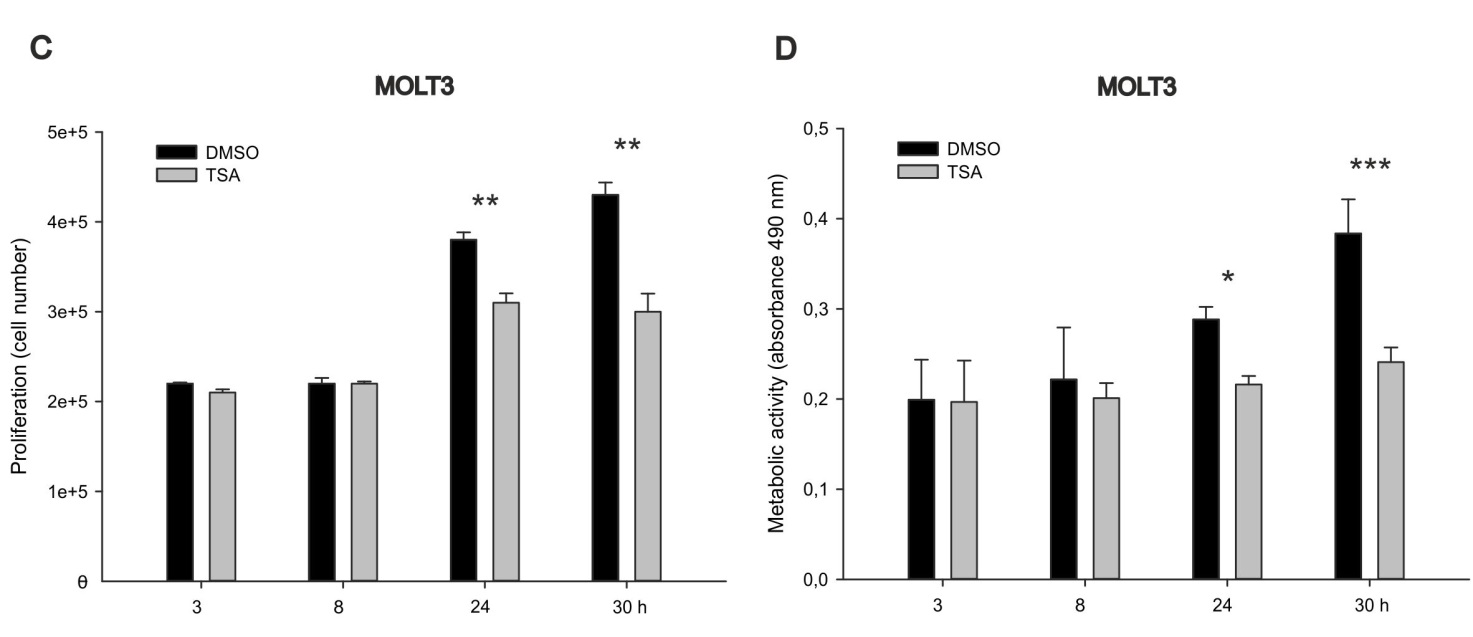
**

**Supplementary figure 1**. **Effects of TSA on apoptosis and proliferation of T-ALL cells.** T-ALL cell lines **(A)** or primary T-ALL cells **(B)** were treated with TSA (0.5 µM) for 24 h. Apoptosis was measured by flow cytometric analysis of Annexin V staining. **(C-D)** MOLT3 cells were treated with TSA (0.5 μM). Cell numbers **(C)** and metabolic activity (MTS assay) **(D)** were assessed at 3, 8, 24 and 30 h from treatment (* P<0.05, ** P<0.01, *** P <0.001, mean ± SD of three independent experiments).

**Suppl. Figure 2**


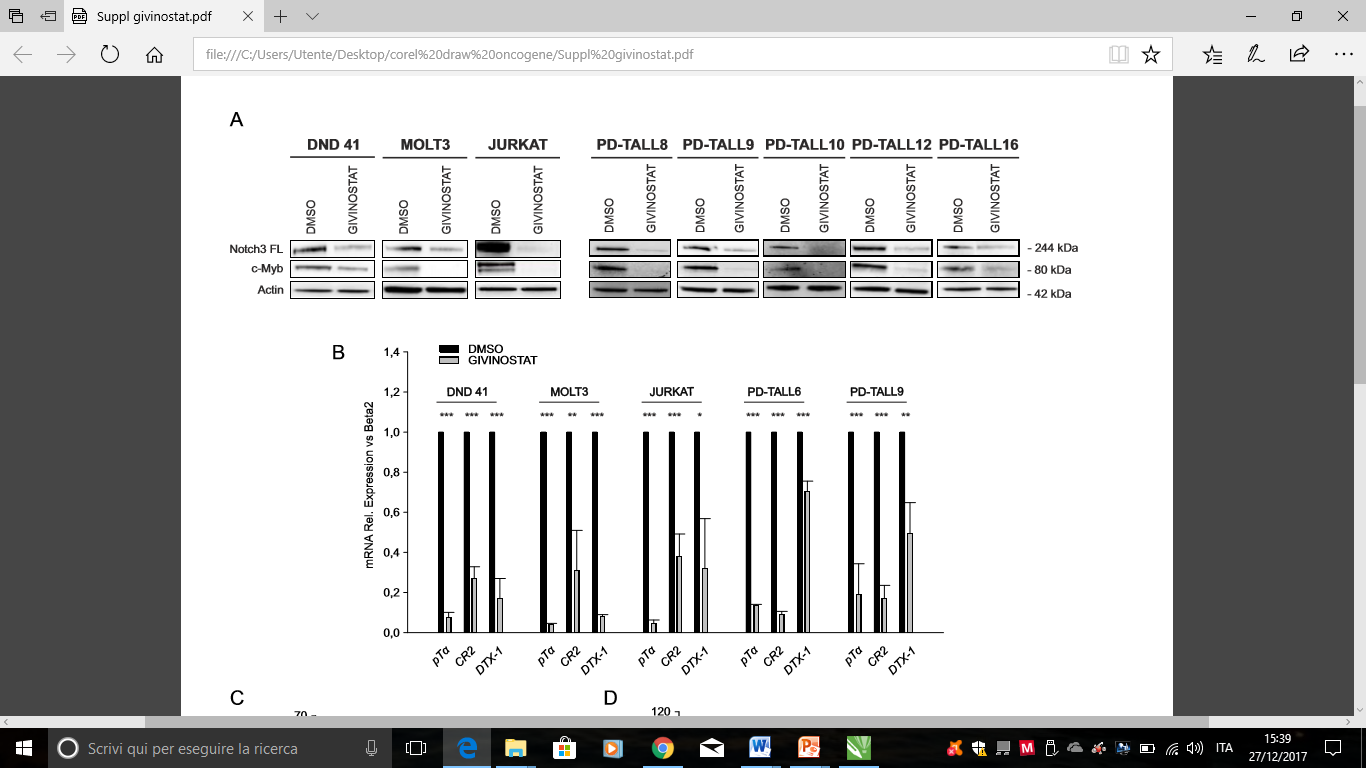


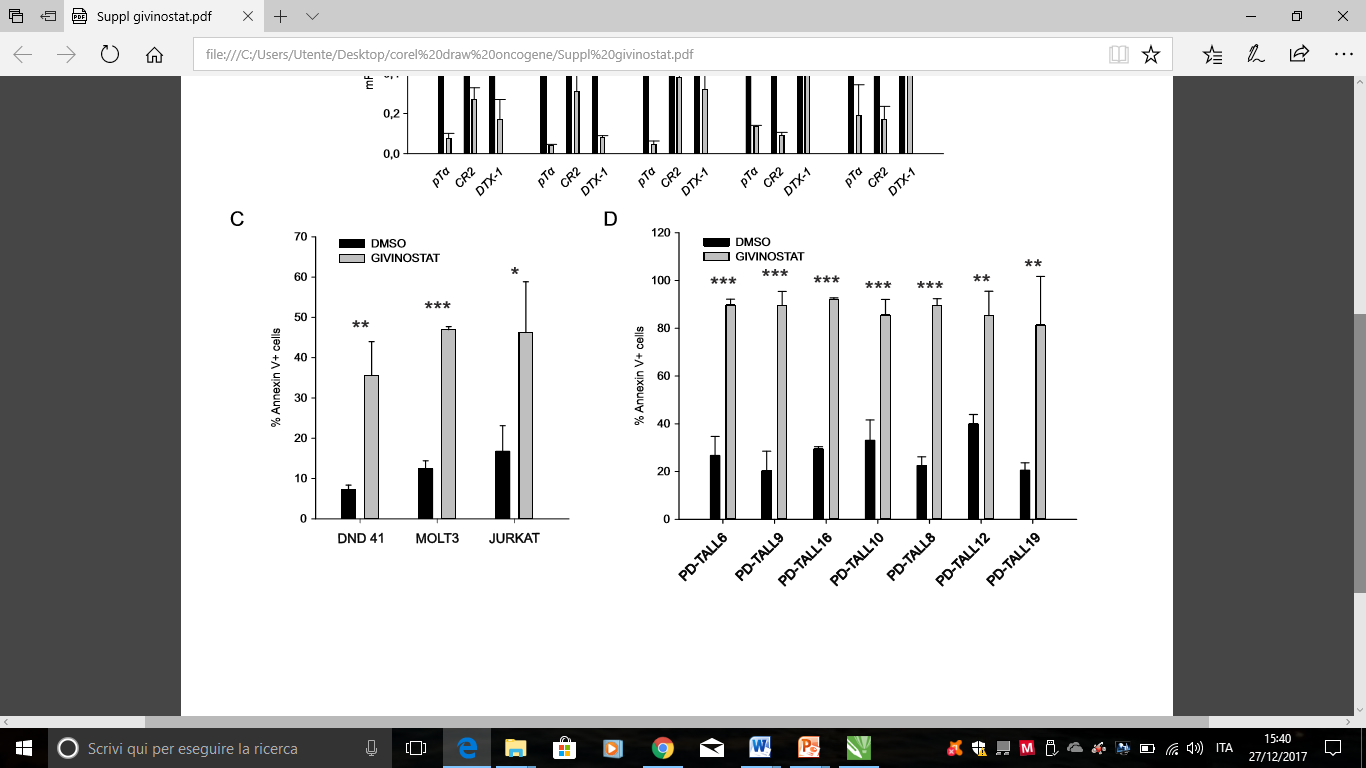


**Supplementary figure 2. Givinostat reduces Notch3 levels and signaling in T-ALL cells and induces apoptosis. (A)** T-ALL cell lines and PDX cells were treated with Givinostat (2 µM) or solvent (DMSO) for 16 h and protein levels analyzed by Western blot. Actin was used as loading control. **(B)** T-ALL samples, including both **c**ell lines and PDX T-ALL cells, were treated with Givinostat for 16 h and mRNA levels of Notch target transcripts (*pTα, CR2, DTX-1*) were analyzed by qRT-PCR. Statistically significant differences are indicated (* P<0.05, ** P<0.01, *** P <0.001, mean ± SD of three independent experiments). T-ALL cell lines **(C)** or primary T-ALL cells **(D)** were treated with Givinostat for 24 h. Apoptosis was measured by flow cytometric analysis of Annexin V staining.

**Suppl. Figure 3**

**
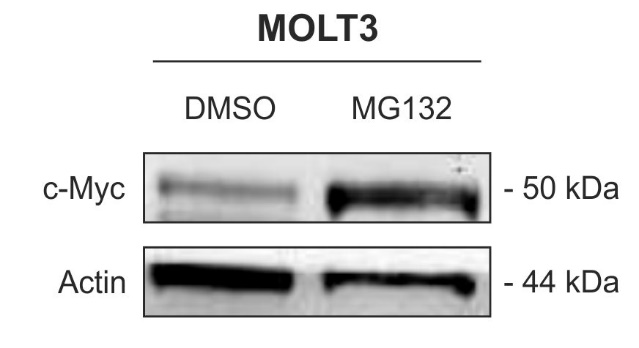
**

**Supplementary figure 3. Assessment of MG132 activity**. MOLT3 were treated with MG132 (20 μM for 8 h) and c-Myc protein levels analyzed by Western blot.

**Suppl. Figure 4**


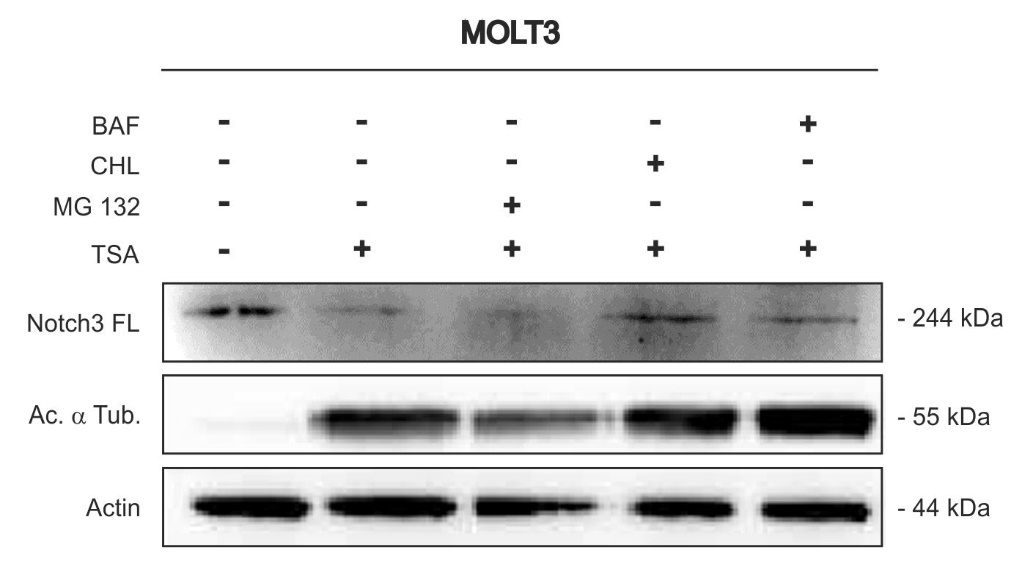


**Supplementary figure 4. The lysosome inhibitor bafilomycin restores Notch3 levels in MOLT-3 cells.** MOLT-3 cells were treated with TSA (0.5 μM) plus MG132 (20 μM) or CHL (20 μM) or bafilomycin (BAF, 100 nM) for 16 h and Western blot analysis was performed.

**Suppl. Figure 5**

**A B**


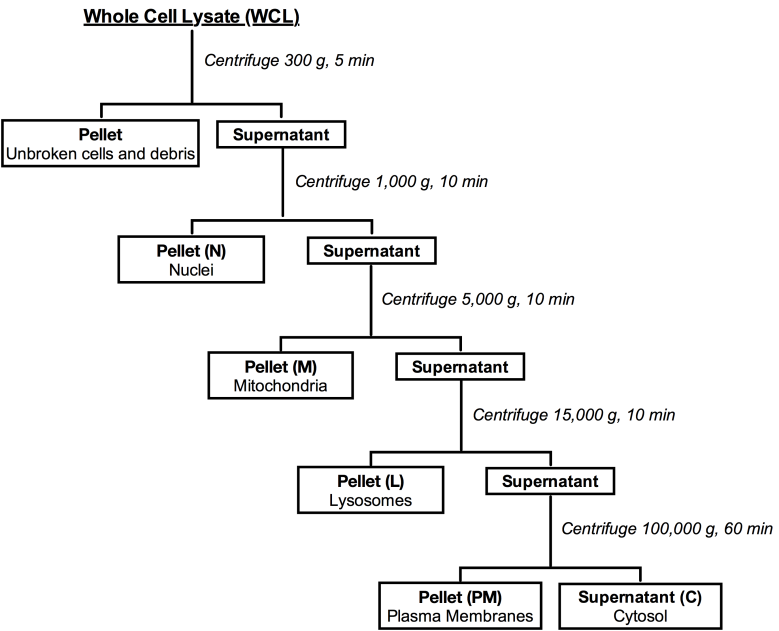
 **
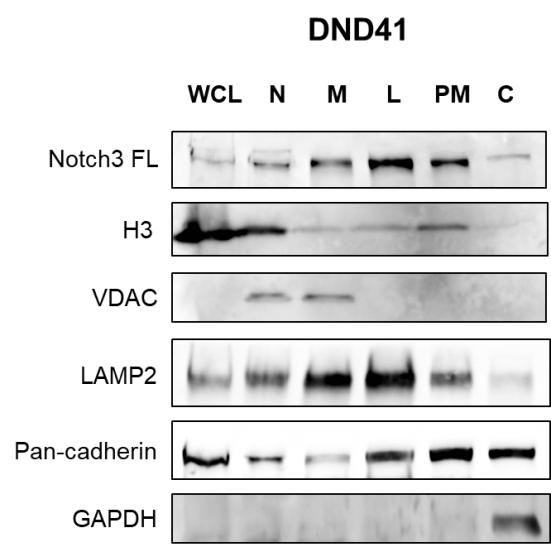
**

**Supplementary figure 5. (A)** A schematic representation of the fractionation protocol used to isolate subcellular fraction in DND 41 cell line. **(B)** 1x10^8^ pelleted parental DND 41 cells underwent subcellular fractionation as described in panel (A). Protein extracts for each fraction were analyzed by western blot. WCL: whole cell lysate; N: nucleus; M: mitochondria; L: lysosome; PM: plasma membrane; C: cytosol. Expression of Notch3 FL fraction by fraction is reported. LAMP2, Pan-cadherin, GAPDH, VDAC, Histone H3 and β-tubulin were used as specific markers for lysosomes, plasma membranes, cytosol, mitochondria, nuclei and total fractions, respectively.

**Suppl. Figure 6**


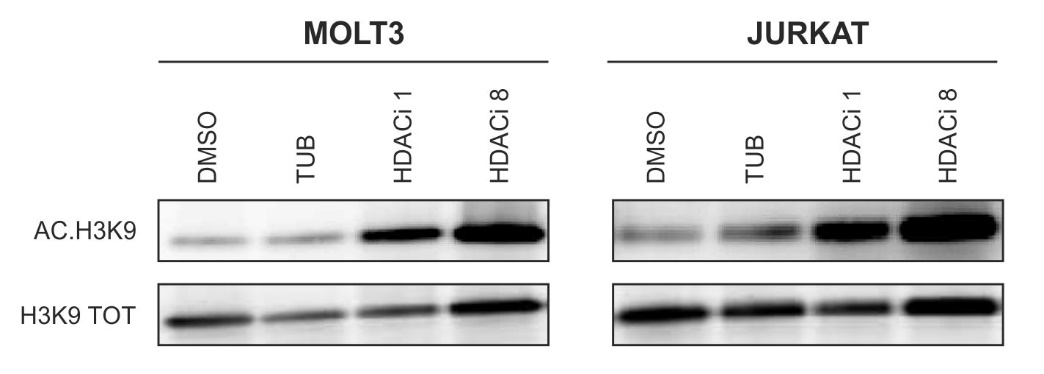


**Supplementary figure 6. Histone acetylation following treatment with class-specific HDACi.** MOLT-3 and Jurkat cells were treated for 16 h with HDAC6 inhibitor tubacin (TUB), HDAC1 inhibitor (HDAC1i), HDAC8 inhibitor (HDAC8i), all used at 2 µM. Protein levels of acetylated histone H3K9 and of histone H3 total were analyzed by Western blot.

**Suppl. Figure 7**

**
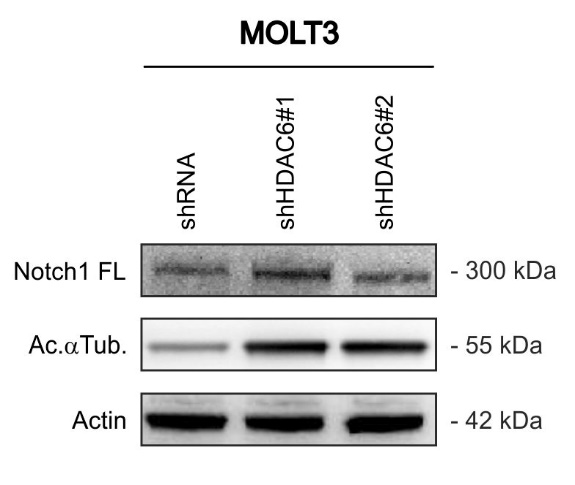
**

**Supplementary figure 7.** MOLT-3 cells were transduced with a lentiviral vector expressing a scramble shRNA or with two different shHDAC6 vectors. Five days after transduction, cells were harvested and western blot analysis of Notch1 FL was performed. Increased *a*-tubulin acetylation confirmed effective inhibition of HDAC6.

**Suppl. Figure 8**

**
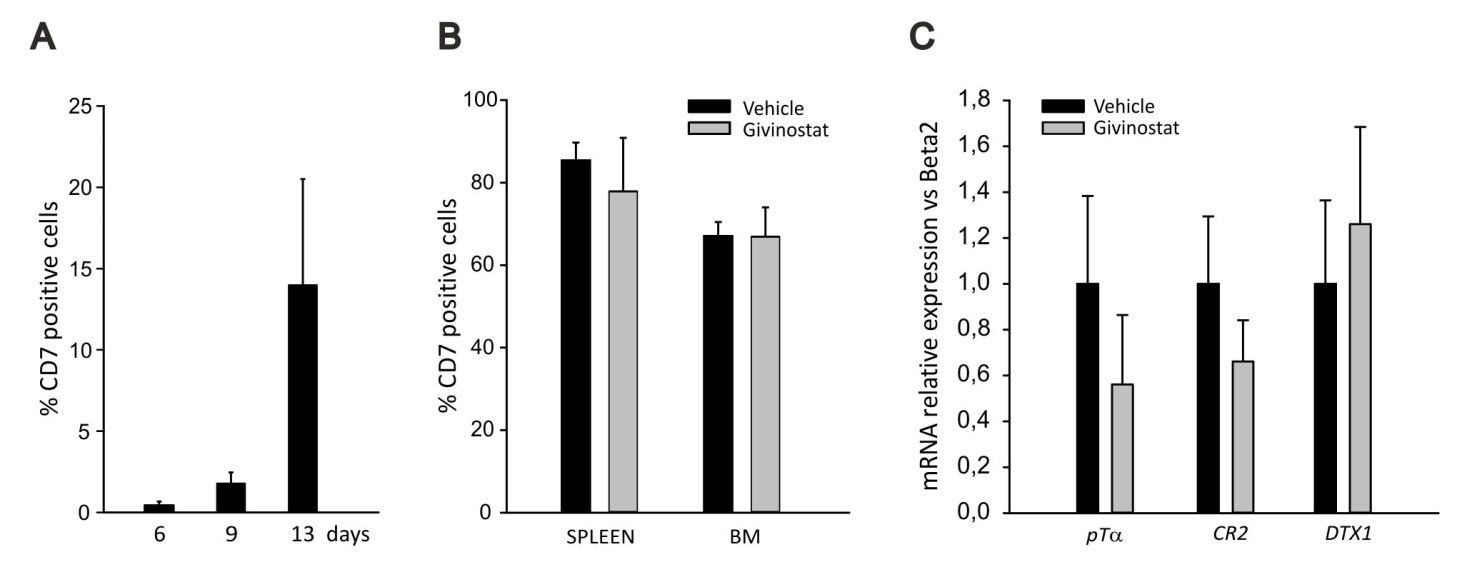
**

**Supplementary figure 8.** Leukemia engraftment of PD-TALL12 cells in NOD/SCID mice was monitored by blood drawings. **(A)** Flow cytometric analysis of the percentage of CD7 positive cells at serial time points (n=5/6 mice/group). (**B**) The histogram represents levels of T-ALL cells in the spleen and bone marrow at day 13, measured by flow cytometry.
